# The authors reply

**DOI:** 10.1016/j.kint.2020.02.006

**Published:** 2020-05

**Authors:** Raina D. Ramnath, Matthew J. Butler, Rebecca R. Foster, Simon C. Satchell

**Affiliations:** 1 Bristol Renal, Translational Health Sciences, Bristol Medical School, University of Bristol, Bristol, UK

We write in response to the letter entitled “There is Little Evidence That the Endothelial Glycocalyx Has a Specific Role in Glomerular Permeability of Albumin,”^1^ in reference to our original publication.^2^ The title of the letter is very misleading. It represents only the opinion of this author and is diametrically opposed to the data presented in our paper and an accumulating body of evidence including *in vivo* multiphoton measurements.^3^ This author’s claims are not supported by experimental observations, and we and others have addressed them extensively including in a response^4^ to a previous similar letter. It is curious that the major focus of the letter seems to be on charge selectivity when charge is not mentioned in our article.
